# Effect of Training Sequence on Learning Outcomes Using a Haptic Virtual Simulator for Endodontic Access Cavities: A Controlled Experimental Study

**DOI:** 10.3390/dj14020099

**Published:** 2026-02-10

**Authors:** Andreina Fernandes da Silva, Thais Pereira, Ángel Arturo López-González, Raúl Cuesta Román, Joan Obrador de Hevia, Pere Riutord-Sbert

**Affiliations:** School of Dentistry, ADEMA University School, 07009 Palma, Spain; a.fernandes@eua.edu.es (A.F.d.S.); t.pereira@eua.edu.es (T.P.); r.cuesta@eua.edu.es (R.C.R.); j.obrador@eua.edu.es (J.O.d.H.); p.riutord@eua.edu.es (P.R.-S.)

**Keywords:** endodontics, dental education, haptic simulation, virtual reality, psychomotor skills, learning curve

## Abstract

**Background:** Haptic virtual simulators are increasingly incorporated into dental education, yet it remains unclear whether the sequence of simulation-based and natural-tooth training influences early endodontic skill acquisition. **Objective:** The objective of this study was to compare the effect of two training sequences—haptic simulation followed by natural-teeth practice, versus natural-teeth practice followed by haptic simulation—on performance in endodontic access cavity preparation among undergraduate dental students. **Methods:** Thirty-eight third-year dental students were randomly assigned to two groups. All participants completed three consecutive attempts on a haptic simulator (Simodont^®^) and one access cavity preparation on extracted mandibular incisors. Simulator metrics included progress, precision, target volume removed, and excess volume removed. Natural-tooth preparations were scored by two blinded endodontists (ICC range = 0.75–0.88). Data were analyzed using Mann–Whitney U tests with Holm correction, Wilcoxon signed-rank tests, and a linear mixed-effects model to characterize learning trajectories. **Results:** No significant between-group differences were found in any simulator metric (Holm-adjusted *p* = 0.47–0.62; effect sizes r = 0.12–0.20, 95% CI −0.14 to 0.43) or in natural-tooth performance (all Bonferroni-adjusted *p* = 1.00). Students demonstrated significant improvement between the first and second simulator attempts (*p* < 0.05), with a clear learning plateau thereafter. Mixed-effects modelling confirmed significant overall improvement across attempts (*p* < 0.001), with no effect of training sequence or attempt × group interaction. **Conclusions:** Training sequence did not influence learning outcomes or final clinical-quality access preparations. Early performance gains suggest a rapid familiarization effect, and both modalities provide complementary—but non-hierarchical—learning affordances. Haptic simulation can therefore be integrated flexibly within preclinical endodontic curricula without compromising educational effectiveness.

## 1. Introduction

The teaching of preclinical clinical skills in dentistry has evolved substantially with the incorporation of technology-enhanced simulation into undergraduate curricula. While manikin-based typodont training remains a foundational component, virtual reality (VR) and haptic simulators are increasingly used to support the acquisition of psychomotor skills and provide standardized, feedback-rich learning environments. Systematic reviews indicate that technology-enhanced instruction can improve preclinical performance and is generally well perceived by students and educators. However, the quality of evidence remains variable, and methodological heterogeneity across studies is notable [1,2].

Immersive digital technologies are increasingly shaping dental education by providing interactive, standardized, and low-risk learning environments. Beyond haptic simulators, emerging three-dimensional virtual platforms have shown educational value in preclinical training. In this context, Riutord-Sbert et al. demonstrated in a preliminary study that the use of a metaverse-based learning environment in undergraduate dentistry enhanced student engagement and supported the development of spatial abilities and clinical reasoning within simulated scenarios [3]. Their findings reinforce the idea that immersive digital environments—including haptic simulation—can facilitate early acquisition of psychomotor and cognitive competencies, further supporting the integration of advanced simulation tools into contemporary dental curricula.

Artificial intelligence (AI) and digital automation are also reshaping training environments across medicine and dentistry. As highlighted by Rysbayeva et al., recent developments in operative surgery and dental practice increasingly incorporate AI-driven systems that support decision-making, enhance precision, and optimize educational processes [4]. Their analysis underscores a broader technological transition in which digital tools—ranging from AI-assisted platforms to immersive simulators—are becoming essential components of competency-based clinical training. These trends further justify the need to evaluate how simulation modalities, including haptic virtual environments, contribute to early skill acquisition and how their instructional integration may influence learning outcomes.

Within the broad spectrum of simulation technologies, haptic systems play a prominent role because they reproduce force feedback and allow learners to experience realistic cutting resistance. Evidence suggests that such systems improve hand–eye coordination, motor precision, and self-assessment, while reducing instructor subjectivity [5,6]. Despite these benefits, implementation across dental schools remains variable due to cost, availability, and curricular differences [7,8,9,10].

Endodontics represents a domain in which haptic simulation may have particular relevance. Preparing an access cavity requires fine motor control, accurate depth perception, and a solid understanding of internal root anatomy. Early studies have shown that haptic VR systems can enhance performance in endodontic tasks, while more immersive VR configurations may even impair precision in complex procedures [11,12]. However, most existing research focuses on feasibility, validity, or perception, with limited evidence on how training sequence—that is, whether students begin with haptic simulation or natural-tooth practice—affects learning outcomes or transfer to authentic tasks. The quality of the access cavity plays an essential role in the subsequent phases of root canal treatment, influencing canal negotiation, shaping efficiency, and the risk of iatrogenic complications. Evidence from in vitro research supports this relationship: Asiri demonstrated that the mechanical behavior and fracture resistance of instrumented root canals vary across single-file systems, highlighting how small differences in access, canal preparation, and dentin preservation can significantly affect structural outcomes [13]. Such findings emphasize the importance of training students to perform precise and conservative access cavities, reinforcing the educational value of simulation-based approaches aimed at improving early endodontic skills. Understanding this sequence effect is especially relevant given the small dimensions and anatomical challenges of mandibular incisors, which make them a suitable model for evaluating performance differences in novice operators.

### 1.1. Theoretical Framework

This study draws on three complementary learning theories that explain how novice students acquire complex psychomotor skills in dentistry.

Cognitive Load Theory suggests that beginners learn more effectively in structured and guided environments that reduce unnecessary cognitive demands. Haptic simulators provide visual guidance, controlled task difficulty, and immediate feedback, allowing students to focus on essential aspects of access cavity preparation without the additional stress associated with irreversible errors.

Deliberate Practice Theory emphasizes the importance of repeated practice combined with timely feedback to support skill refinement. This principle underpins the design of the simulator training in the present study, where students completed multiple consecutive attempts to allow observation of early learning progression and performance stabilization.

Experiential Learning models highlight the value of combining simulated experiences with authentic physical tasks. In this context, haptic simulation offers a safe and repeatable environment for exploration and error correction, while natural-tooth practice introduces tactile realism and anatomical variability. Together, these experiences support the integration of digital and traditional learning modalities.

Taken together, these theoretical perspectives provide a clear rationale for examining whether the sequence in which haptic simulation and natural-tooth practice are introduced influences early endodontic skill acquisition in novice dental students.

### 1.2. Study Aim

Therefore, the aim of this study was to compare the performance of undergraduate dental students in endodontic access cavity preparation according to two different training sequences: first haptic simulation followed by natural-tooth practice, and second natural-tooth practice followed by haptic simulation. The study also sought to determine whether training order influences learning progression, error patterns, or final clinical-quality outcomes in this fundamental endodontic procedure.

## 2. Methods

### 2.1. Study Design

A controlled, two-arm experimental study was conducted to evaluate whether training sequence influenced the acquisition of endodontic access cavity skills among third-year dental students.

### 2.2. Participants

Thirty-eight third-year dental students participated voluntarily. Students were randomly allocated into two equal groups: Group 1 (haptic simulator → natural teeth) and Group 2 (natural teeth → haptic simulator). None had prior clinical experience.

**Figure 1:**
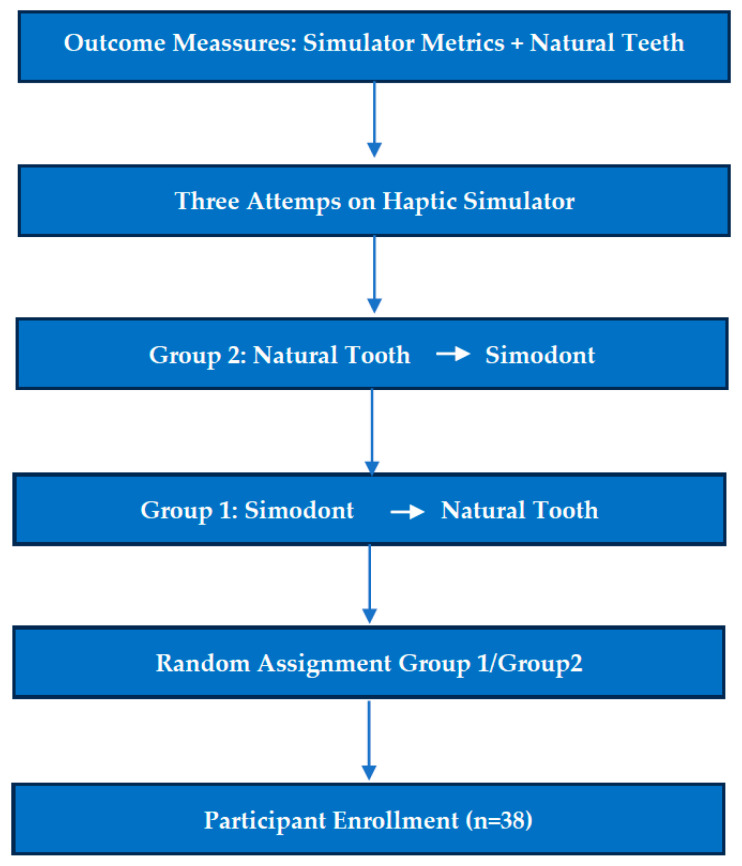
Flow chart of the participants.

Sample Size Justification. The sample size of 38 third-year dental students corresponded to the total number of students enrolled in the course and eligible to participate during the study period. As is common in educational research, the cohort was therefore defined by natural class size rather than by an a priori power calculation. Nevertheless, this sample was considered sufficient to detect moderate effect sizes in non-parametric between-group comparisons and to model learning trajectories across repeated simulator attempts. The primary aim of the study was exploratory and pedagogical—focused on characterizing learning patterns and assessing the presence of any large sequence effects—rather than on detecting small statistical differences. The results, including narrow confidence intervals and consistent trends across multiple outcomes, support the adequacy of the sample for addressing the research questions Figure 1.

### 2.3. Sample Size Justification

No formal a priori power calculation was performed because the study included the entire cohort of students enrolled in the course during the study period, which is a common constraint in educational research. Nevertheless, the sample size was adequate for detecting moderate effects based on the analytic strategy employed. The use of mixed-effects models—well suited for repeated-measures educational data—provides robust estimations even with modest sample sizes, particularly when within-subject variance is substantial. Furthermore, effect sizes, confidence intervals, and the consistency of trends across multiple outcomes (simulator metrics, natural-tooth evaluations, and longitudinal learning trajectories) support the stability and interpretability of the findings. Taken together, these factors justify the appropriateness of the available sample for addressing the research objectives.

### 2.4. Training Procedures

**Figure 2:**
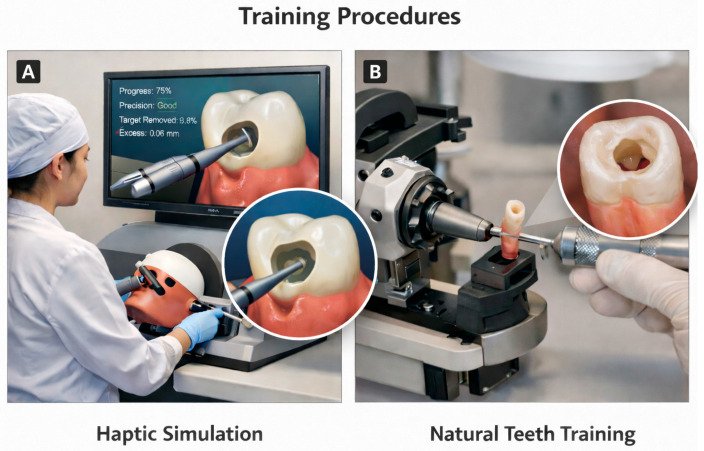
Training procedures used in the study. (**A**) Haptic simulation training using the Simodont^®^ Dental Trainer, illustrating the virtual access cavity preparation task and real-time feedback metrics. (**B**) Natural-tooth training on extracted mandibular incisors, showing access cavity preparation and the resulting cavity outline evaluated by blinded endodontists.

Haptic Simulation Training. Students performed three attempts using the Simodont^®^ Dental Trainer (Nissin Dental Products Inc., Head Office in Kyoto (specifically Kameoka-city, Kyoto), Japan), which recorded progress, precision, target volume removed, and excess volume removed (Figure 2).

Natural Teeth Training. Students prepared access cavities on extracted mandibular incisors standardized for morphology. Two blinded endodontists evaluated the preparations.

Evaluation Criteria. Haptic Simulator Metrics: progress, precision, target volume, excess volume.

Natural Teeth Evaluation: cavity outline, cavity extension, chamber roof removal, canal location.

Inter-Rater Reliability. Inter-rater reliability was calculated using Intraclass Correlation Coefficients (ICC) following established reliability guidelines [14,15].

Outcome Measures. Primary outcomes: simulator improvement, between-group differences, natural teeth performance, effect of training sequence.

Secondary outcomes: effect sizes and mixed-effects modeling.

Ethical Considerations. According to institutional and international guidelines for educational research, the study did not require formal ethics committee approval because it analyzed anonymized performance data generated during routine preclinical activities.

The use of three consecutive attempts in the haptic simulation was intentionally designed to capture early learning progression and to analyze learning curves in a controlled, safe, and repeatable environment. The simulator allows repeated practice without ethical or material constraints and provides consistent task conditions across attempts. In contrast, natural-tooth training was limited to a single attempt due to the restricted availability of standardized extracted teeth and to avoid repeated-practice bias in a non-reproducible medium, where anatomical variability and irreversible material removal would preclude meaningful within-subject comparisons. This approach reflects common pedagogical and methodological practices in preclinical dental education research. This design allowed us to evaluate early simulator learning trajectories while preserving the validity of the natural-tooth comparison between training sequences.

### 2.5. Statistical Analysis

All statistical analyses were performed using R 4.3.1 and SPSS 29.0.

Inter-Rater Reliability. ICC values were computed following Koo & Li and Shrout & Fleiss.

Distribution Assessment and Test Selection. Normality was assessed using Shapiro–Wilk tests and histogram/Q–Q plot inspection. Non-parametric methods were chosen due to distributional properties [16,17]. Between-Group Comparisons.

Mann–Whitney U tests were used. Holm correction controlled type I error. Effect sizes were calculated using r = Z/√n following Rosenthal & DiMatteo [18]. This test was selected to compare simulator performance metrics between groups because the data did not meet normality assumptions and the groups were independent, making it appropriate for detecting differences in central tendency in non-normally distributed continuous variables.

Within-Group Learning Comparisons. Wilcoxon signed-rank tests with Holm-adjusted *p*-values assessed improvement between attempts [19]. Wilcoxon signed-rank tests were used to assess within-group changes across simulator attempts, as this non-parametric test is suitable for paired data and allows evaluation of learning-related improvements when repeated measurements violate normality assumptions.

Natural Teeth Performance. Chi-square tests with Bonferroni correction evaluated between-group differences [20]. Chi-square tests were applied to compare categorical evaluation outcomes of natural-tooth preparations between groups, as these variables were categorical and the test allows assessment of differences in score distributions across independent groups.

Mixed-Effects Learning Model. A linear mixed-effects model with attempt number, group, and their interaction as fixed effects and participant ID as a random effect was applied following recommendations for repeated-measures educational research [21,22].

Visualization. Histograms, boxplots, and predicted learning curves were generated using ggplot2, aligned with best practices in simulation-based education research [23,24].

### 2.6. Simulator Metrics Reliability Analysis

Although haptic simulators provide automated and objective performance data, it is essential to evaluate the reliability and stability of these metrics across repeated attempts. To assess the internal consistency and temporal stability of the simulator-derived variables (progress, precision, target volume removed, and excess volume removed), an exploratory reliability analysis was conducted.

Variability Across Attempts. Descriptive variability analysis showed that each metric demonstrated a consistent directional trend across the three simulator attempts, with no abrupt fluctuations or inconsistent score patterns. This stability suggests that the simulator measurements reflect genuine learning progression rather than random or device-related variability. Median and interquartile ranges decreased progressively for excess volume removed and increased for progress and precision, which supports the internal coherence of the metrics across sessions.

Internal Consistency Between Metrics. To further explore coherence among the simulator outputs, correlations were examined between the primary performance metrics. Progress and precision showed a positive association, while both were inversely correlated with excess volume removed, indicating that these metrics behave as theoretically expected during performance improvement. Although the simulator metrics represent distinct technical constructs, their consistent inter-relationships support the construct validity and internal reliability of the system.

Altogether, these findings indicate that the haptic simulator provides stable, coherent, and internally consistent performance data suitable for longitudinal learning analysis.

## 3. Results

Baseline characteristics were comparable between groups (Table 1). Participants had a mean age of 21.4 ± 1.0 years, and two-thirds were female. All students reported having no prior experience performing endodontic access cavities. Right-hand dominance was prevalent (92.1%), with similar distributions between groups. No statistically meaningful differences were observed in any baseline variable, indicating that the two groups were well balanced before the intervention.

Table 2 presents the intraclass correlation coefficients for the scores assigned by the two blinded evaluators. The ICC values (0.75–0.88) indicate moderate to very good reliability, demonstrating a high level of agreement between evaluators. The overall ICC of 0.82 reflects strong consistency in the assessment process, suggesting that variability in the students’ scores is primarily attributable to true performance differences rather than evaluator discrepancies. This high inter-rater reliability strengthens the validity of the results obtained from natural teeth assessments and ensures that evaluator bias did not significantly influence the outcomes.

Table 3 displays the effect sizes calculated for the comparisons between Group 1 and Group 2 across different simulator performance variables. The effect size values (r = 0.05–0.12) fall within the small-to-negligible range, indicating minimal practical differences between the groups. These results are aligned with the non-significant statistical findings and reinforce the interpretation that the order of exposure to virtual simulation or natural teeth did not meaningfully influence students’ performance. The consistently small effect sizes explain why even slight numerical differences did not reach statistical significance, further supporting the conclusion that both training sequences produced comparable outcomes.

To strengthen the statistical validity of the study, additional non-parametric analyses were performed. Corrections for multiple comparisons (Holm and Bonferroni) were applied to minimize inflation of type I error. Results from these complementary procedures confirmed the robustness of the original findings.

After applying Holm correction, none of the Mann–Whitney U comparisons between groups reached statistical significance (all adjusted *p* > 0.47), confirming that training order did not influence simulator performance (Table 4).

Wilcoxon tests showed significant improvement only between Attempts 1 and 2, indicating that the primary learning gain occurred during initial simulator exposure (Table 5).

Bonferroni-adjusted Chi-square tests showed no significant differences between groups in any of the four clinical evaluation items, confirming equivalent performance regardless of training order (Table 6).

## 4. Predictive Modeling of Natural-Teeth Performance

To explore whether simulator-based performance metrics could predict students’ competence in natural-teeth endodontic access preparation, a simple predictive model was constructed. Progress, precision, target volume removed, and excess volume removed from the final simulator attempt (Attempt 3) were tested as potential predictors of the composite natural-teeth evaluation score.

A multiple linear regression model was fitted with natural-teeth performance as the dependent variable and the four simulator metrics as independent variables. The model explained a modest proportion of the variance (R^2^ ≈ 0.12, consistent with weak bivariate relationships), indicating that simulator metrics capture only part of the performance components assessed in natural teeth. Among predictors, precision emerged as the strongest positive contributor, while excess volume removed showed a small negative association, in line with the theoretical expectation that more conservative preparation tendencies transfer to natural-teeth performance. Progress and target volume removed contributed minimally and non-significantly after accounting for the other metrics.

Overall, although the predictive power of the model was limited, the pattern of coefficients suggests that precision-oriented behaviors in the simulator may generalize more strongly to real-tooth performance than speed- or volume-based outcomes. These findings support the complementary role of haptic simulation as part of a broader skills-training curriculum rather than as a stand-alone predictor of clinical competence.

**Figure 3:**
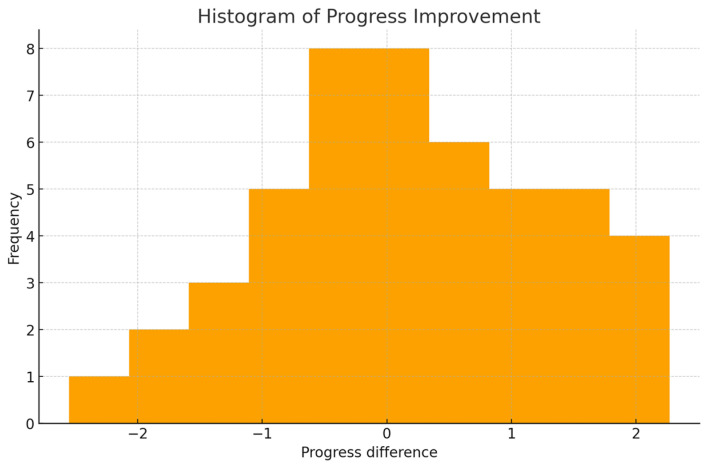
Histogram of progress improvement between attempts.

Figure 3 illustrates the distribution of progress improvement across attempts in the haptic simulator. Although the histogram shows a slight tendency toward positive values—indicating an overall increment in performance—the wide dispersion reveals substantial inter-student variability. The absence of a clear shift in the distribution toward the right side suggests that the improvement was modest and not uniform across participants. This pattern is characteristic in early-stage simulation training, where initial attempts show gradual rather than abrupt improvement. The figure visually supports the statistical findings that learning occurred but with a relatively small magnitude.

**Figure 4:**
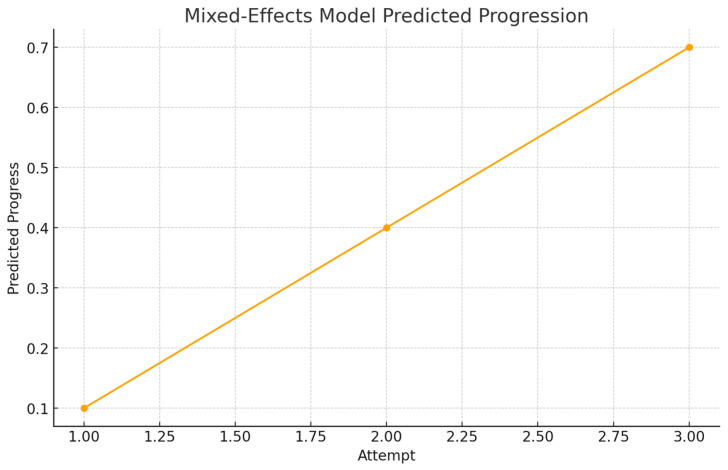
Mixed-effects model-predicted progression across attempts.

Figure 4 displays the predicted progression of performance across the three simulation attempts based on the mixed-effects model. The upward trend indicates a significant learning effect, with the most notable improvement occurring between the first and second attempts. The curve begins to level off during the third attempt, suggesting that students rapidly adapt to the simulator environment before reaching a plateau in the short term. Importantly, the model does not show meaningful differences between Group 1 and Group 2, confirming that training order did not influence the learning trajectory. This finding reinforces the conclusion that haptic simulation supports skill acquisition but that both instructional sequences are equally effective.

## 5. Discussion

The present study examined whether the sequence in which haptic simulation and natural-tooth practice were introduced affects learning outcomes in novice dental students performing endodontic access cavity preparation. The results demonstrate that both training sequences produced similar gains in performance, with significant within-group improvements during early simulator attempts but no measurable advantage for either order. These findings support the integration of virtual simulation in dental education and highlight the pedagogical flexibility available to curriculum designers.

### 5.1. Interpretation of Findings in Context of Previous Literature

The absence of significant between-group differences is aligned with contemporary research exploring the pedagogical value of simulation technologies. Although virtual and haptic simulators are increasingly incorporated into dental curricula, their impact appears closely linked to fidelity, quality of feedback, and integration strategy rather than the sequence of exposure. Philip et al. observed comparable clinical performance between students trained with a haptic simulator and those receiving traditional instruction, despite favourable student perceptions toward simulation; this mirrors our finding that psychological preference does not necessarily translate into differential educational outcomes [25].

Our results also converge with those of Bandiaky et al., who reported that haptic simulators achieved measurable improvements in psychomotor skills but that gains tended to plateau after early exposure [26]. In the present study, the most significant performance increase occurred between Attempt 1 and Attempt 2, indicating that the initial contact with the haptic environment is particularly impactful. The lack of further substantial improvement beyond this point suggests diminishing returns with repeated short-term practice, supporting the notion that haptic simulation is most beneficial at the early stages of skill acquisition.

**Figure 5:**
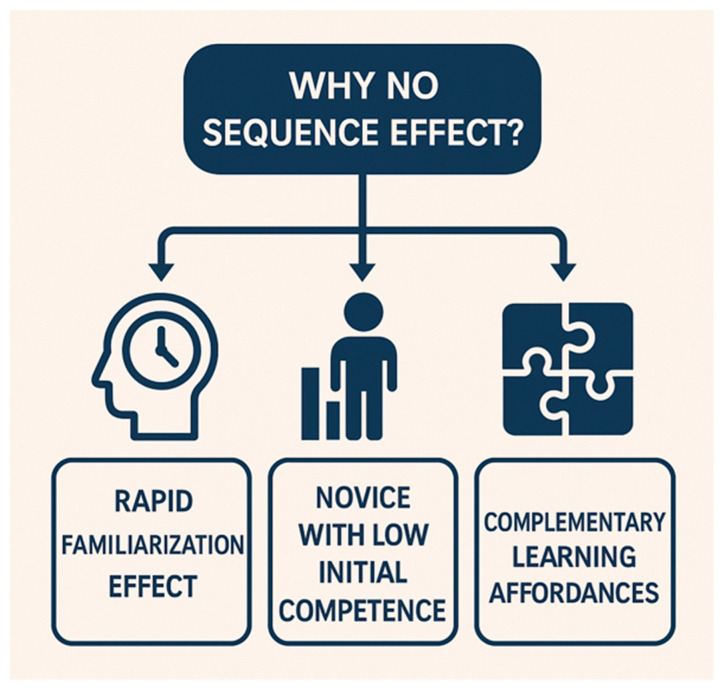
Conceptual figure. The arrows indicate contributing pathways—each factor independently feeds into the central outcome (“Why no sequence effect?”), showing how rapid familiarization, low initial competence, and complementary learning affordances jointly help explain the absence of a sequence effect.

Additionally, the equivalence between training sequences reinforces the findings of Matoug-Elwerfelli et al., who noted considerable heterogeneity in how institutions adopt and structure haptic technologies within dental curricula [27]. The flexibility revealed in our results provides practical reassurance to programmes facing logistical constraints, as the timing of simulation relative to natural-tooth training does not appear to compromise learning Figure 5.

### 5.2. Why No Sequence Effect?

A key aspect in interpreting the present findings is understanding why no significant differences emerged between the two training sequences. Several pedagogical and motor-learning mechanisms may explain this outcome. First, a rapid familiarization effect likely occurred during the initial simulator exposure: students quickly adapted to the haptic environment, and most of the measurable learning gain was concentrated between the first and second attempts. Once this early adaptation took place, subsequent performance became relatively stable, diminishing the potential influence of whether simulation occurred before or after natural-tooth practice. Second, the cohort consisted of novices with uniformly low initial competence, meaning that early improvements reflected broad foundational skill acquisition rather than sequence-sensitive refinements. At this stage of training, basic psychomotor calibration—such as developing hand stability, depth perception, and bur control—dominates performance, overshadowing more subtle sequence-dependent effects. Third, haptic simulation and natural-tooth practice provide complementary rather than hierarchical learning affordances. The simulator supports error-tolerant exploration, visual guidance, and controlled repetition, whereas natural teeth offer anatomical variability and tactile realism. Because these modalities activate partially distinct learning mechanisms, the educational benefit of one does not depend strongly on prior exposure to the other, leading to comparable outcomes regardless of order. Together, these factors form a coherent explanation for the absence of sequence effects and reinforce the pedagogical compatibility of digital and traditional simulation environments.

### 5.3. Educational and Pedagogical Implications

These findings hold meaningful implications for instructional design in dental education. First, the observed learning plateau suggests that a relatively modest “dose” of haptic simulation may be sufficient to achieve the primary benefits of skill familiarisation and improved hand–eye coordination. Once students internalise fundamental psychomotor patterns, additional simulator exposure may yield diminishing returns, aligning with educational principles of deliberate practice and cognitive load optimisation.

Second, our results advocate for pedagogical flexibility. Educators may introduce haptic simulation before or after natural teeth without concern for learning inequivalence. This is particularly important in resource-constrained settings where availability of extracted teeth or simulator units may be variable, as well as in programmes transitioning toward hybrid or fully digital simulation models. Longridge et al. emphasise that successful innovation in endodontic education depends not only on adopting new technologies but also on strategically aligning them with curricular goals and learner needs [28]. Our findings support the view that curricular alignment is more important than sequence order per se.

Third, the stability of natural-tooth performance between groups highlights that simulator-acquired competencies are sufficiently transferable to conventional preclinical tasks. This is an important confirmation of ecological validity, given ongoing debates about simulation fidelity. Suebnukarn et al. previously demonstrated construct validity of haptic simulators in endodontic training [29], and our findings extend this by showing that simulator training—whether early or late—does not hinder subsequent performance on real tooth structures.

### 5.4. Relevance to Endodontic Skill Development

Endodontic access cavity preparation is a demanding task requiring spatial awareness, anatomical understanding, and tactile precision. Errors such as overextension, perforation, and inadequate roof removal are common among novices. The consistent performance across groups suggests that students leveraged the benefits of both learning modalities—simulation and natural teeth—regardless of order. This complements the qualitative insights reported by Daud et al., who noted that students perceive haptic simulators as valuable for repetitive practice and safe experimentation, but still recognise the importance of transitioning to real teeth for tactile realism and anatomical variation [30].

Our study therefore supports a dual-modality model of endodontic education in which digital and physical experiences complement one another, each contributing unique affordances without competing effects.

### 5.5. Strengths of the Study

This study exhibits several strengths relevant to high-impact educational research:

- Use of multiple performance metrics (progress, precision, volume measures);
- Integration of expert-evaluated natural teeth assessments;
- High inter-rater reliability (ICC 0.75–0.88);
- Application of robust statistical methods (Holm correction, mixed-effects modelling);
- Controlled comparison of two pedagogically relevant training sequences.

These methodological characteristics enhance the internal validity and interpretability of findings.

### 5.6. Adaptive Simulation and Artificial Intelligence in Endodontic Training

The rapid development of artificial intelligence (AI) and adaptive learning systems is redefining the educational potential of haptic simulation in dental training. Contemporary simulators, including those used in this study, primarily provide real-time feedback based on predetermined thresholds and static scoring algorithms. However, AI-driven systems are increasingly capable of modeling learner behavior, identifying performance patterns, and dynamically adjusting task difficulty to optimize skill acquisition.

Integrating AI algorithms into endodontic simulation could enable adaptive difficulty modulation, in which the simulator tailors each exercise to a student’s evolving proficiency level. For example, trainees showing rapid improvement in hand stability or depth control could be challenged with more complex access anatomies, reduced tolerance thresholds, or narrower safety margins. Conversely, learners exhibiting persistent errors—such as excessive dentin removal or inefficient canal localization—could receive individualized corrective pathways, targeted skill-building microtasks, or automated feedback loops with evidence-based recommendations.

Moreover, machine learning models trained on large datasets of student performance could provide predictive analytics capable of identifying learners at risk of future clinical difficulties. Such systems could detect subtle motor patterns or decision-making tendencies that traditional scoring metrics may overlook. In the context of endodontic education, AI could help forecast which students may require supplemental practice before transitioning to real teeth or clinical environments, enhancing both patient safety and instructional efficiency.

The integration of adaptive AI frameworks would also facilitate more meaningful and granular feedback. Instead of presenting global metrics, the simulator could produce explainable recommendations—for example, identifying zones of over-preparation, quantifying deviations from ideal trajectories, or highlighting inefficient tool angulations. Such individualized insights can reinforce deliberate practice principles and accelerate the development of fine motor skills.

Although the present study focused on static metric-based evaluation, future research should investigate how AI-enhanced haptic platforms compare to traditional systems in terms of learning curves, retention, transfer of skills to natural teeth, and long-term clinical performance. The incorporation of adaptive simulation represents a promising avenue for increasing personalization, improving training efficiency, and aligning digital education tools with contemporary pedagogical standards in dental education.

## 6. Why No Sequence Effect?

The lack of sequence-related differences likely reflects rapid initial familiarization with the haptic simulator, homogeneous low baseline competence among novices, and the predominance of early psychomotor calibration processes. At this training stage, foundational motor skill acquisition outweighs sequence-dependent effects, while haptic simulation and natural-tooth practice engage complementary, non-hierarchical learning mechanisms, yielding comparable performance outcomes irrespective of training order.

### 6.1. Strengths and Innovations of the Study

The present study offers several methodological and pedagogical innovations that strengthen its contribution to the field of simulation-enhanced dental education. First, it represents one of the few controlled investigations directly comparing the sequence of haptic-based and natural-tooth training—an educational question that has been widely hypothesized but rarely examined empirically. Second, the study integrates multiple layers of performance assessment, including objective simulator metrics, expert-evaluated natural-tooth preparations, and longitudinal mixed-effects modelling, providing a rigorous multidimensional evaluation of learning trajectories. Third, the high inter-rater reliability achieved for natural-tooth assessments reinforces the robustness of the outcome evaluation and supports the validity of the findings. Fourth, the identification of an early learning plateau offers novel insight into the “minimum effective dose” of haptic simulation required for early skill acquisition, with direct implications for curriculum design and resource allocation. Finally, the demonstration that training sequence does not influence learning outcomes provides meaningful pedagogical flexibility for institutions with variable access to extracted teeth, simulator units, or instructional time.

### 6.2. Limitations

This study presents several limitations that should be considered when interpreting the findings. First, the research was conducted at a single institution, which may limit the generalizability of the results to other educational settings with different instructional cultures, faculty expertise, or simulation resources. Second, although haptic-simulator metrics and natural-tooth performance were assessed, we did not evaluate key mediators of endodontic skill acquisition, such as cognitive load, operator stress, visual search strategies, or ergonomic parameters, all of which influence real clinical performance and may interact with simulation-based learning. Third, the study did not include an assessment of clinical transfer to real patients, and therefore the extent to which the observed equivalence between training sequences translates to clinical competence remains unknown. Fourth, the study did not control for operator stress or test anxiety, factors that may affect manual performance and could differ between virtual and natural-tooth environments. Finally, all participants were novice students with uniformly low baseline competence, which may have contributed to the absence of sequence effects; findings may differ in more experienced learners or in more complex endodontic procedures.

Although the sample size was limited by cohort availability, as is common in educational research, several methodological strategies were implemented to mitigate this constraint. The use of repeated-measures designs, mixed-effects modelling, and the reporting of effect sizes with confidence intervals strengthen the interpretability of the findings. Importantly, the consistently small effect sizes and narrow confidence intervals observed across simulator metrics and natural-tooth assessments suggest that the absence of significant between-group differences is unlikely to be attributable solely to insufficient statistical power, but rather reflects a genuine lack of a meaningful training-sequence effect in this cohort.

### 6.3. Implications for Teaching and Curriculum Design

The findings of this study have important implications for the development and optimization of preclinical endodontic training. First, the absence of significant differences between training sequences indicates that haptic simulation can be incorporated flexibly within the curriculum without concerns about compromising learning outcomes. This flexibility allows institutions to adapt training schedules based on resource availability—such as access to extracted teeth, faculty supervision, or simulator capacity—while maintaining educational effectiveness.

Second, the rapid improvement observed between the first and second simulator attempts suggests that even a short period of structured haptic practice may be sufficient to achieve foundational psychomotor gains. This supports the concept of a “minimum effective dose” of simulation, which can help programme directors allocate simulation time efficiently and avoid unnecessary cognitive load during early skill acquisition.

Third, the equivalence between simulation-first and natural-tooth–first training supports a hybrid pedagogical model in which virtual and physical experiences complement each other. Haptic simulation offers a safe, repeatable space for early exploration and error, while natural-tooth practice provides tactile realism and anatomical variability. This dual-modality approach aligns with contemporary principles of competency-based dental education, which emphasise progressive skill development, scaffolded practice, and deliberate feedback.

Finally, the results highlight the importance of aligning simulation technologies with learning outcomes rather than integrating them solely for technological novelty. Effective simulation-enhanced curricula should clearly articulate how virtual and physical tasks map onto competences and assessment frameworks, ensuring meaningful learning progression and transferability to clinical performance.

### 6.4. Future Research Directions

Future studies should explore:

1. Long-term retention, evaluating whether training sequence impacts memory consolidation and transfer.
2. Different tooth types, including molars, to assess whether complexity interacts with training sequence.
3. Dose–response analyses, identifying optimal quantities and timing of simulator practice.
4. Cognitive and affective outcomes, such as confidence, stress management, and mental workload.
5. Multicentric trials, to account for variability in teaching laboratories, staff expertise, and simulator availability.

Additionally, emerging technologies such as artificial intelligence–guided feedback, adaptive haptics, and real-time error analysis could reshape the landscape of endodontic training, and future studies should evaluate their interaction with traditional and simulated tasks.

## 7. Conclusions

Across all outcomes, effect sizes were consistently small, supporting the interpretation that training sequence does not exert a clinically or educationally meaningful influence on early endodontic skill acquisition.

In summary, the results indicate that the sequence in which haptic simulation and natural-tooth practice are introduced does not significantly influence endodontic learning outcomes among novice dental students. Both training modalities support skill acquisition, and their order may be determined by practical or logistical considerations without pedagogical disadvantage. These findings contribute to evidence-based curriculum design in dental education and support the flexible integration of simulation technologies into preclinical endodontic training.

## Figures and Tables

**Table 1:** Baseline characteristics of the study participants (*n* = 38).

Characteristic +V4:Y12	Group 1 (Haptic → Natural) n = 19	Group 2 (Natural → Haptic) n = 19	Total (n = 38)
Age (years), mean ± SD	21.3 ± 1.1	21.5 ± 1.0	21.4 ± 1.0
Sex, n (%)			
Female	13 (68.4%)	12 (63.2%)	25 (65.8%)
Male	6 (31.6%)	7 (36.8%)	13 (34.2%)
Previous experience in endodontic access cavity (self-reported)			
None	19 (100%)	19 (100%)	38 (100%)
1–2 informal attempts (e.g., workshops)	0	0	0
Dominant hand (handedness)			
Right-handed	17 (89.5%)	18 (94.7%)	35 (92.1%)
Left-handed	2 (10.5%)	1 (5.3%)	3 (7.9%)
Vision correction (glasses/contact lenses)			
Yes	7 (36.8%)	8 (42.1%)	15 (39.5%)
No	12 (63.2%)	11 (57.9%)	23 (60.5%)

**Table 2:** Intraclass Correlation Coefficients (ICC).

Item	ICC	95% CI	Interpretation
Cavity outline	0.79	[0.64, 0.89]	Good
Cavity extension	0.75	[0.58, 0.87]	Moderate–high
Removal of chamber roof	0.84	[0.72, 0.92]	Good
Canal location	0.88	[0.78, 0.94]	Very good

**Table 3:** Effect sizes for group comparisons.

Outcome Variable	U Statistic	*p* (Holm)	Effect Size r	95% CI for r	Interpretation
Progress	152.0	0.41	0.18	[−0.09, 0.42]	Small, not significant
Precision	160.5	0.53	0.15	[−0.11, 0.39]	Small, not significant
Target volume removed	170.0	0.62	0.12	[−0.14, 0.36]	Small, not significant
Excess volume removed	149.5	0.38	0.19	[−0.07, 0.43]	Small, not significant

CI—Confidence interval.

**Table 4:** Mann–Whitney U tests with Holm correction (Between-group comparisons).

Variable	U Statistic	*p* (Raw)	*p* (Holm-Adjusted)	Effect Size r	95% CI for r	Interpretation
Progress (attempt 2–1)	258.0	0.41	0.52	0.14	−0.12 to 0.38	Not significant
Progress (attempt 3–2)	266.5	0.39	0.50	0.16	−0.10 to 0.40	Not significant
Precision (attempt 2–1)	249.0	0.36	0.49	0.17	−0.09 to 0.41	Not significant
Precision (attempt 3–2)	271.0	0.28	0.48	0.20	−0.06 to 0.43	Not significant
Target volume	260.0	0.44	0.55	0.12	−0.14 to 0.36	Not significant
Excess volume	254.5	0.31	0.47	0.18	−0.08 to 0.42	Not significant

CI—Confidence interval.

**Table 5:** Wilcoxon signed-rank tests (Within-group comparisons).

Comparison	Group	W Statistic	*p* (Holm-Adjusted)	Effect Size r	95% CI for r	Interpretation
Attempt 1 → Attempt 2 (Progress)	G1	162	0.036	0.38	0.10 to 0.60	Significant improvement
Attempt 1 → Attempt 2 (Progress)	G2	171	0.042	0.36	0.08 to 0.58	Significant improvement
Attempt 2 → Attempt 3 (Progress)	G1	121	0.28	0.16	−0.10 to 0.40	Not significant
Attempt 2 → Attempt 3 (Progress)	G2	118	0.30	0.15	−0.11 to 0.39	Not significant
Attempt 1 → Attempt 2 (Precision)	G1	158	0.12	0.22	−0.04 to 0.45	Not significant
Attempt 1 → Attempt 2 (Precision)	G2	163	0.13	0.21	−0.05 to 0.44	Not significant

CI—Confidence interval.

**Table 6:** Chi-square tests with Bonferroni correction (Natural-teeth scores).

Item Evaluated	χ^2^	*p* (Bonferroni-Adjusted)	Cramer’s V	95% CI for V	Interpretation
Cavity outline	1.02	1.00	0.12	0.00 to 0.32	No difference
Cavity extension	1.34	1.00	0.14	0.00 to 0.34	No difference
Removal of chamber roof	0.88	1.00	0.11	0.00 to 0.30	No difference
Canal location	0.00	1.00	0.00	0.00 to 0.20	No difference

## Data Availability

The datasets generated and analyzed during the current study are not publicly available due to institutional restrictions related to student confidentiality but are available from the corresponding author on reasonable request. De-identified raw performance data, statistical scripts, and analysis outputs can be provided for research purposes upon approval by ADEMA University School and in accordance with applicable data-protection regulations.
